# Pesticide Side Effects in an Agricultural Soil Ecosystem as Measured by *amoA* Expression Quantification and Bacterial Diversity Changes

**DOI:** 10.1371/journal.pone.0126080

**Published:** 2015-05-04

**Authors:** Louise Feld, Mathis Hjort Hjelmsø, Morten Schostag Nielsen, Anne Dorthe Jacobsen, Regin Rønn, Flemming Ekelund, Paul Henning Krogh, Bjarne Westergaard Strobel, Carsten Suhr Jacobsen

**Affiliations:** 1 Geological Survey of Denmark and Greenland (GEUS), Department of Geochemistry, Copenhagen, Denmark; 2 University of Copenhagen, Department of Biology, Section of Terrestrial Ecology, Copenhagen, Denmark; 3 University of Aarhus, Department of Bioscience, Section of Soil Fauna Ecology and Ecotoxicology, Silkeborg, Denmark; 4 University of Copenhagen, Department of Plant and Environmental Sciences, Section of Environmental Chemistry and Physics, Frederiksberg, Denmark; Universidad de Salamanca, SPAIN

## Abstract

**Background and Methods:**

Assessing the effects of pesticide hazards on microbiological processes in the soil is currently based on analyses that provide limited insight into the ongoing processes. This study proposes a more comprehensive approach. The side effects of pesticides may appear as changes in the expression of specific microbial genes or as changes in diversity. To assess the impact of pesticides on gene expression, we focused on the *amoA* gene, which is involved in ammonia oxidation. We prepared soil microcosms and exposed them to dazomet, mancozeb or no pesticide. We hypothesized that the amount of *amoA* transcript decreases upon pesticide application, and to test this hypothesis, we used reverse-transcription qPCR. We also hypothesized that bacterial diversity is affected by pesticides. This hypothesis was investigated via 454 sequencing and diversity analysis of the 16S ribosomal RNA and RNA genes, representing the active and total soil bacterial communities, respectively.

**Results and Conclusion:**

Treatment with dazomet reduced both the bacterial and archaeal *amoA* transcript numbers by more than two log units and produced long-term effects for more than 28 days. Mancozeb also inhibited the numbers of *amoA* transcripts, but only transiently. The bacterial and archaeal *amoA* transcripts were both sensitive bioindicators of pesticide side effects. Additionally, the numbers of bacterial *amoA* transcripts correlated with nitrate production in N-amended microcosms. Dazomet reduced the total bacterial numbers by one log unit, but the population size was restored after twelve days. The diversity of the active soil bacteria also seemed to be re-established after twelve days. However, the total bacterial diversity as reflected in the 16S ribosomal RNA gene sequences was largely dominated by Firmicutes and Proteobacteria at day twelve, likely reflecting a halt in the growth of early opportunists and the re-establishment of a more diverse population. We observed no effects of mancozeb on diversity.

## Introduction

Molecular-based methods have a great potential to provide sensitive, specific and cost-efficient measurements that are suitable for the evaluation of pesticide side effects on important soil ecosystem functions as well as the microbial community composition. Molecular analyses based on directly extracted DNA or RNA allow measurements of bacterial or fungal diversity of either the total or active communities, respectively, and of specific functions, e.g., via the quantification of genes or gene transcripts that are involved in nitrogen turnover. Currently, the only standard analyses, which provide the basis for hazard assessment of pesticides to soil microorganisms, are carbon and nitrogen transformation tests [1]. These tests measure the general microbial activity as reflected in glucose-induced respiration and the nitrification activity as reflected the production of nitrate [2,3].

Nitrification is very stress-sensitive, and several pesticides inhibit the process [4–6]. Nitrification is a central step in the nitrogen cycle, where ammonia oxidizers first oxidize ammonia into nitrite in a rate-limiting step that is catalyzed by ammonia monooxygenase, after which nitrite oxidizers use nitrite oxidoreductase to oxidize nitrite into nitrate [7]. It was recently discovered that in addition to the well-known ammonia-oxidizing β- and γ-Proteobacteria, some archaea also contain a variant of ammonia monooxygenase and perform ammonia oxidation [8,9].

A large number of studies have exploited the genetic variation in *amoA* genes to distinguish and quantify the number of bacterial and archaeal variants of the genes under different agricultural management and soil environment conditions [10–14]. These studies have demonstrated a heterogeneous distribution of ammonia oxidizers, in particular higher pH and nitrogen availability favor bacteria over archaea [15]. Based on the apparent niche differentiation between the two groups, Wessén and Hallin suggested qPCR quantification of archaeal and bacterial *amoA* gene copies as a potential biomarker to monitor the soil ecosystem and the response to land use and environmental conditions [16].

The quantification of *amoA* mRNA transcripts, in addition to *amoA* gene copy numbers, yields direct information of the actual gene expression of the soil system. In contrast to DNA, mRNA transcripts are highly labile messenger molecules that are only present during periods of gene expression. Therefore, an abundance of specific mRNA transcripts potentially correlates better with enzyme activity and therefore process rates than their DNA counterpart. Previously, Bælum and co-workers demonstrated this relationship for the mineralization of the pesticide MCPA, which correlated well with the number of *tfdA* transcripts but not with the gene copies in the soil [17]. Here, we evaluate the hypothesis that the quantification of bacterial and archaeal *amoA* transcripts is a sensitive tool to predict the immediate effects of pesticides on soil nitrification.

Soil bacterial diversity is important in maintaining the wide functional potential of the soil ecosystem. Thus, pesticide risk assessment should include an analysis of the microbial diversity in the soil. Because of the high bacterial species richness in the soil, Wertz and co-workers claimed that some diversity reduction will not affect major functions, such as carbon mineralization and nitrogen turnover [18]. Other researchers, however, have demonstrated that soil functioning will change at reduced diversity levels. Griffiths and co-workers reported lower nitrification, denitrification and methane oxidation under decreased biodiversity [19], and Yang and co-workers found a decreasing biological fractionation of ^13^C with decreasing microbial diversity, reflecting the shorter food-chains [20]. Finally, several studies have demonstrated that the ability of the soil system to resist invasion by new microorganisms decreases with decreasing species richness [21,22].

Methods to describe the bacterial diversity as affected by pesticides have until recently been limited to the small fraction of the bacterial community that is culturable [23]. Methods that analyze the total extracted DNA or RNA from the soil allow a more inclusive analysis. In a recent review, Jacobsen and Hjelmsø recorded no studies that reported the application of next-generation sequencing techniques to assess the pesticide effects on the soil microbial diversity [24].

In this study, we evaluate the pesticide effects using two novel molecular techniques: 1) an assay based on reverse-transcription qPCR, which quantifies the abundance of microbial functional gene transcripts, and 2) an assay evaluating the diversity of bacterial communities. For functional genes, we quantified *amoA* transcripts from both bacteria and archaea, and for diversity, we evaluated the 454 amplicon sequencing of the V3 region of the 16S ribosomal RNA (rRNA) gene—in both cases in response to treatment with the pesticides mancozeb (Tridex DG) or dazomet (Basamid GR).

## Materials and Methods

### Soil

The permission for soil sampling was granted from the Askov Experimental Station under the Dept. of Agroecology, Aarhus University, Denmark. The soil was a loamy sand (37% coarse sand, 42% fine sand, 10% silt, 9% clay and 2% organic matter) that was sampled from an agricultural field (55°28'20''N, 09°06'36''E) at the Askov Experimental Station, Denmark. The soil contained 1.6% total C, 0.14% total N, 19.9 mg/kg NO_3_^-^-N, and 1.53 mg/kg NH_4_^+^-N and had a pH of 6.4 and a C:N ratio of 11.4. We sieved the soil (4-mm mesh) and kept it in the dark at 5°C until the beginning of the experiment.

### Pesticides

We tested two formulated pesticides, Basamid GR and Tridex DG. Basamid GR is a soil disinfectant that is used to control a range of soil pests including soil nematodes, fungi and weeds. Basamid GR contains the active compound dazomet (tetrahydro-3,5-dimethyl-2H-1,3,5-thiadiazine-2-thione) and was obtained as granulate from Kanesho Soil Treatment (Brussels, Belgium). Tridex DG is a fungicide with the active compound mancozeb [[1,2-ethanediylbis[carbamodithioato]](2-)] manganese mixed with [[1,2-ethanediylbis[carbamodithioato]](2-)] zinc. Tridex DG was obtained as granulate from Cerexagri, Inc. (Plaisir Cedex, France). The concentrations that were used in the experiments corresponded to the recommended application dose for Basamid GR (266 mg/kg dry soil) and to five times the recommended application dose for Tridex DG (13.3 mg/kg dry soil). These doses were calculated, assuming a soil density of 1.5 g cm^-1^ and a field distribution of the pesticides in the top 5 cm of the soil.

### Microcosm set-up

The soil was air dried at room temperature in the dark for three days and then homogenized by sieving (2 mm mesh). We prepared microcosms with 10 g of sieved soil in 50 ml polypropylene tubes. We added pesticide and/or ammonium sulfate (100 mg N/kg soil) that had been dissolved in 2 ml of sterile distilled H_2_O to a final soil moisture content of 60% WHC. We set up a full factorial design with six treatments with either dazomet or mancozeb or no pesticide in every combination with or without ammonium sulfate. All of the microcosms were covered with polyvinylchloride film and incubated in the dark at 20°C until sampling after one hour and after 3, 7, 12, 21 and 28 days (on day 7, only nitrate was measured). At each sampling point, we processed three microcosms from each treatment. A subsample of 500 mg of soil was flash-frozen in liquid nitrogen for the later extraction of DNA and RNA, and the remaining soil sample was stored at -20°C until nitrate determination.

### Nitrate determination

Nitrate was extracted according to the OECD 216 guidelines [2] by mixing 9.5 g of moist soil with KCl (0.1 M) to a total volume of 47.5 ml, with the modification that the extraction tubes were shaken manually until all of the soil particles were suspended before being placed on an end-over-end shaker for 1 hour according to the guidelines.

The nitrate concentrations of the soil extracts were determined via a chromogenic microplate method according to Hood-Nowotny et al. [25]. The nitrate concentration was determined in duplicate for each soil extract (diluted to 0.2–2.0 mg/l N).

### Extraction of nucleic acids and cDNA synthesis

We extracted DNA and RNA simultaneously from the soil using a phenol-chloroform extraction procedure [26] with the following modifications: to minimize the DNA loss by sorption to clay particles, we added 0.5 ml of G2 (GEUS, Copenhagen, DK, US patent application no. 20120094353) to 1.4 mm ceramic bead tubes (Mo Bio Laboratories, Inc., Carlsbad, CA) and freeze-dried the tubes prior to use. We added hexadecyltrimethylammonium bromide (CTAB) and phenol chloroform to the bead tubes along with the frozen soil samples and performed bead beating at speed 5 for 20 s in a FastPrep FP120 (BIO101, Farmingdale, NY). Two runs of bead beating were performed with an intermediate cooling step for one minute on ice. The aqueous phase was separated by centrifugation for ten minutes (16,000 *g*) at 4°C. After removing the phenol with chloroform-isoamyl alcohol, 1 μl of glycogen (Roche, Basel, Switzerland) was added with 800 μl of 30% polyethylene glycol, and the samples were placed on ice for two hours to facilitate nucleic acid precipitation. The samples were kept on ice throughout the extraction.

We purified the RNA/DNA extracts using the NucleoSpin RNA Clean-up XS kit (Macherey-Nagel, GmbH & Co. KG, Düren, Germany) and eluted them in 20 μl of RNase-free H_2_O; the samples were subsequently split into two subsamples. One subsample was diluted tenfold with RNase-free H_2_O and stored at -80°C until DNA analysis. The other subsample was immediately subjected to DNase-treatment using the RTS DNase Kit (Mo Bio) according to the manufacturers’ instructions. We used the DNase-treated sample as the template in cDNA production using random hexamer primers (Thermo Scientific, Inc., Vilnius, Lithuania) and the RevertAid Premium RT kit (Thermo Scientific) in a RT-PCR procedure according to the manufactures’ protocol. The extraction, DNase treatment and reverse transcriptase were performed in one working day. The quantity and quality of the extracted RNA was determined on an Agilent 2100 Bioanalyzer using the prokaryote total RNA pico chip, which uses the relative signal intensity of 16S and 23S rRNA to calculate the RNA integrity number (RIN).

### Real-time PCR assays

We quantified the number of *amoA* gene copies and mRNA transcripts by real-time PCR using the primers amoA-1F and amoA-2R [27] for bacterial ammonia oxidizers and amoA19F [11] and CrenamoA616r48x [28] for archaeal ammonia oxidizers. These primers generate a PCR amplification product of 491 bp for bacterial *amoA* and 440 bp for archaeal *amoA*, respectively. The PCR reactions contained 1 × SYBR Premix Ex Taq (Tli RNaseH Plus) (Takara Bio, Inc.), 5 pmol each primer and 1 μl of DNA or cDNA template in a final volume of 20 μl. The amplification was performed in an iCycler Thermocycler (Bio-Rad Laboratories, USA) at 95°C for 1 min followed by 40 cycles of 95°C for 30 s, 58/55°C (bacterial/archaeal *amoA*) for 30 s and 72°C for 45 s. As standard for bacterial *amoA* quantification, we PCR-amplified the *amoA* gene of the *Nitrosomonas europaea* ATCC19718-derived lux-marker strain (pHLUX20) [29] using the primers amoA-1F and amoA-2R and cloned it into the *E*. *coli* pCR 2.1-TOPO vector (Invitrogen, Carlsbad, CA). We used the fosmid clone 54d9 [9] as the standard for the archaeal *amoA*. We generated standard curves from the extracted plasmids using tenfold dilutions of 10^1^ to 10^7^ bacterial *amoA* copies per microliter and 3×10^1^ to 3×10^6^ archaeal *amoA* copies per microliter.

We quantified the number of total bacterial 16S rRNA and rRNA gene copies using the primers 341F (5′-CCTAYGGGRBGCASCAG-3’) and 806R (5′-GGACTACNNGGGTATCTAAT-3’) [30], yielding an amplification product of 466 bp. The PCR reactions were carried out in a final volume of 20 μl containing 1 × SsoFast EvaGreen Supermix (Bio-Rad), 0.4 μM each forward and reverse primer, 1 mg/ml BSA (BIORON GmbH, Ludwigshafen, Germany) and 1 μl of DNA template. The amplification was performed in a CFX96 Real-Time System (Bio-Rad) at 98°C for 15 min and 35 cycles of 98°C for 30 s, 56°C for 30 s and 72°C for 30 s followed by a final elongation step at 72°C for 7 min and a high-resolution melting curve. As a standard, we used tenfold dilutions of DNA from *Escherichia coli* K-12 containing seven 16S rRNA gene copies per genome [31].

Negative controls of the DNase-treated RNA samples were included in the qPCR to ensure the absence of contaminating DNA. The specificity of the PCR amplification was tested via the inspection of the melting curves that were prepared at the end of each PCR run. A subset of the PCR products was also run on a gel to verify the presence of a single band of the correct size.

### 454 sequencing of bacterial 16S rRNA and rRNA genes

We selected the DNA and cDNA samples from the non-treated soil on day 0 and from soil from every treatment on day twelve for sequencing. Day twelve was chosen because the high-resolution melting curves that were obtained after qPCR amplification of the 16S rRNA genes showed the largest difference between the pesticide-treated and non-treated soil on this day [32]. The DNA and cDNA were PCR-amplified using 0.4 units of Phusion Hot Start II DNA Polymerase (Thermo Scientific), 1 × Phusion HF buffer (Thermo Scientific), 4 nmol dNTP mixture, 10 pmol each 341F and 806R, 1 μl of DNA or cDNA template (10 × diluted) and H_2_O to a total volume of 20 μl. The PCR conditions were 98°C for 30 s, followed by 30 cycles of 98°C for 5 s, 56°C for 20 s and 72°C for 20 s, and a final extension at 72°C for 5 min. The PCR products were run on a 1.25% agarose gel, and specific bands with the expected size of 466 bp were excised and purified using the Montage DNA Gel Extraction Kit (Millipore, Billerica, MA). Tags were added in a second PCR using identical primers but with 10-bp-long individual attached MIDs (Roche) and only 15 PCR cycles. The tagged PCR products were then re-purified, and the DNA concentrations were measured on a Qubit (Invitrogen). Finally, the samples were mixed, creating an equimolar solution with a total of 1 μg DNA. The adapter-ligation, emulsion PCR and 454 sequencing were performed by Beckman Coulter Genomics (Brea, CA) on a 454 GS FLX Titanium Plate (Roche) (1/2 titanium plate).

We used the QIIME V.1.6 bioinformatics tool to analyze the 454 sequences [33]. The reads with a quality score less than 25 in a sliding window of 50 bp were removed from the dataset. We applied the Denoise algorithm [34] to reduce the errors that are introduced by sequencing. Chimeric sequences were removed with the script Chimera Slayer [35]. The sequences were then mapped to a reference 16S rRNA gene database (Greengenes V.12.10). Singletons were deleted to remove the artificial sequences that were not picked up by the Denoise algorithm. Taxonomic tables and rarefaction curves were constructed from the remaining sequences (~2500 per sample). The rarefaction curves did not reach equilibrium; therefore, we could not determine the absolute species number. Therefore, we randomly selected 980 sequences from each sample to compare the species richness between the samples.

### Fate studies

We analyzed the mineralization and sorption of mancozeb in the soil by ^14^C-labeling and radioactivity measurements. The Institute of Isotopes Co. (Budapest, Hungary) delivered the labeled mancozeb, which had a specific activity of 64.16 mCi/g. Mancozeb was labeled in a position so that ^14^C was not released until the compound was completely mineralized, and the last daughter compound ethylenurea (EU) degraded to CO_2_. We set up triplicates in 100 ml airtight flasks with 20 g of soil with or without ammonium sulfate (100 mg N/g soil) and water regimes corresponding to 60% WHC. Tridex was added to the soil at 2.7 mg/kg and ^14^C-labeled mancozeb at 10,000 DPM per flask. A base trap containing 2 ml of NaOH was placed in each flask to collect ^14^CO_2_. During the two months of incubation at 20°C, we replaced the base traps regularly. We measured the samples on a Perkin Elmer Tri-Carb 2810 TR scintillation counter using 10 ml of Wallac OptiPhase HiSafe 3 scintillation cocktail (Perkin Elmer, Turku, Finland) per sample.

We measured the sorption of mancozeb in the soil as a function of the pesticide concentration using a procedure that was modified from the OECD guidelines [36]. The sorption isotherms were determined by setting up triplicate samples of 1 g of soil in glass vials with Teflon caps. Initially, the soil was equilibrated with 1 ml of a 0.01 M CaCl_2_ solution for twelve hours. Suspensions of Tridex and ^14^C-labeled mancozeb were subsequently added to the vials to reach a 1:10 soil-to-solution ratio at final concentrations of 0, 0.2, 0.4, 1.0, 2.0 and 4.0 mg of Tridex per liter and 2500 DPM per ml. The vials were mixed on an end-over-end rotator for two hours or 24 hours at room temperature and thereafter centrifuged at 1500 *g* for ten minutes. The supernatant was then transferred to 2 ml Eppendorf tubes and centrifuged again at 10,000 *g* for ten minutes. Non-adsorbed mancozeb was determined by mixing 1 ml of supernatant with 10 ml of Wallac OptiPhase HiSafe 3 scintillation cocktail and measuring radiation by liquid scintillation counting.

The fumigant dazomet degrades rapidly in moist soil by chemical conversion to methyl isothiocyanate (MITC) [37,38]. We measured the volatilization of MITC via the application of ^14^C-labeled MITC (American Radiolabeled Chemicals, Missouri, USA) to moist (60% WHC) or air-dried soil in airtight serum flasks with Teflon stoppers. The flasks were incubated at 20°C, and the volatilization was measured over a 72 hour period. Subsamples were redrawn from the headspace using a hypodermic needle and injected into new airtight flasks with scintillation liquid. The radioactivity of MITC in the gas phase was counted in a gas-liquid equilibrium.

### Statistics

We analyzed the effects of pesticides and ammonium sulfate on the gene copies, mRNA transcripts and nitrate production using the SAS Enterprise Guide 4.1 interface of the SAS 9.1 package (Statistical Analysis System Institute, 2002–2003). We used three-way ANOVAs with pesticides and ammonium sulfate as the qualitative variables and time as the quantitative variable. The data were log-transformed before analysis to obtain the variance homogeneity.

## Results

### Quantification of *amoA* transcripts and genes

**Fig 1:**
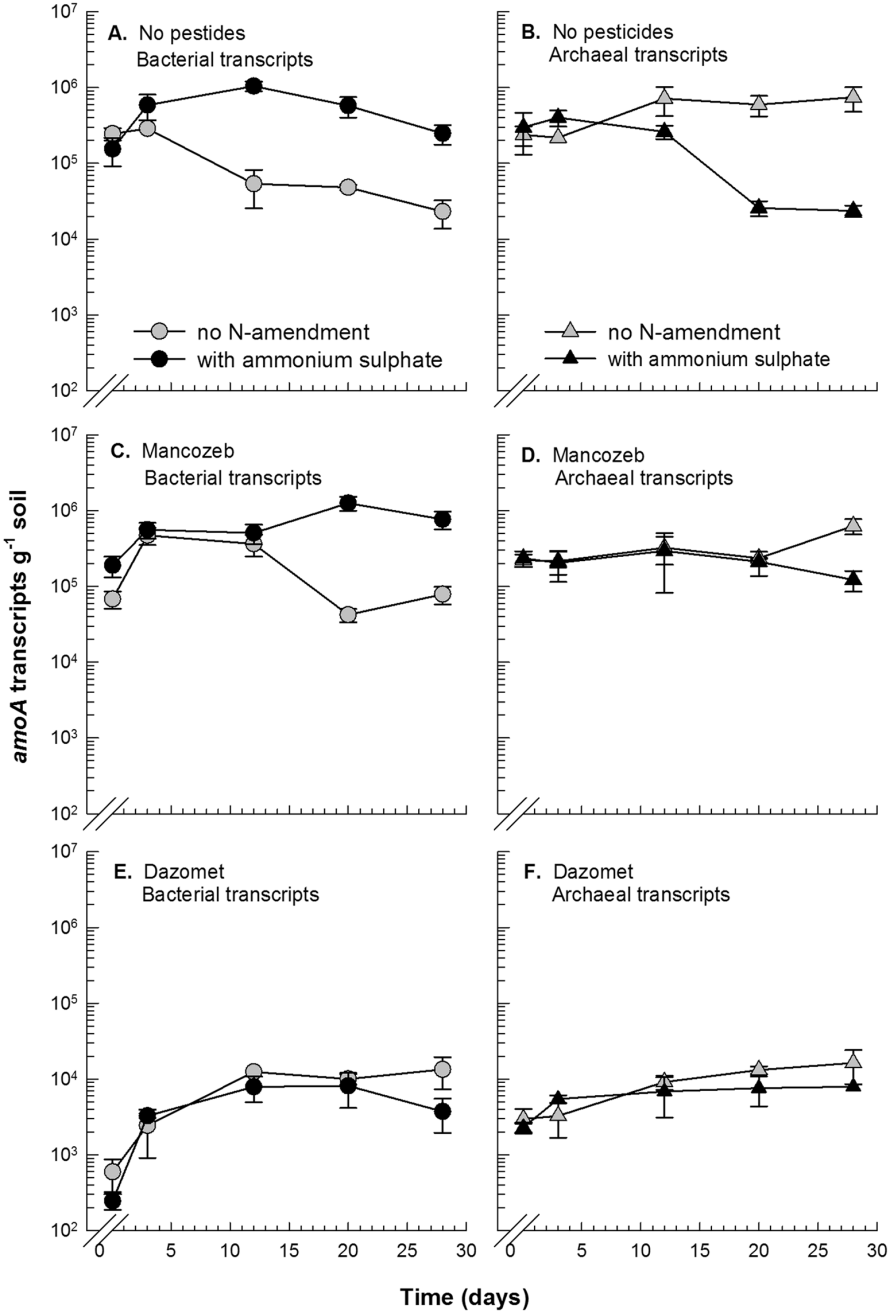
Quantification of *amoA* transcripts by RT-PCR. Abundance of *amoA* transcripts for bacteria (A+C+E) and for archaea (B+D+F) in treatments without pesticides (A+B) and in treatments with mancozeb (C+D) and dazomet (E+F). In each plot, the number of transcripts is shown in treatments without N amendment and with the amendment of ammonium sulfate. The depicted values are the means of triplicate samples, and the error bars indicate standard error. Note that the first data point in each plot indicates measurements one hour after pesticide exposure.

The number of *amoA* transcripts decreased significantly in response to dazomet (P<0.0001). One hour after exposure, the bacterial *amoA* transcripts were reduced by 400 to 1000-fold, and the archaeal *amoA* transcripts were reduced by 100-fold compared with the treatments without pesticides (Fig 1A, 1B, 1E and 1F). Three days after the dazomet treatment, a significant (P<0.001) recovery was observed, showing a tenfold increase in the bacterial and a doubling in the archaeal *amoA* transcripts in the soil that was amended with ammonium sulfate (Fig 1E and 1F).

**Fig 2:**
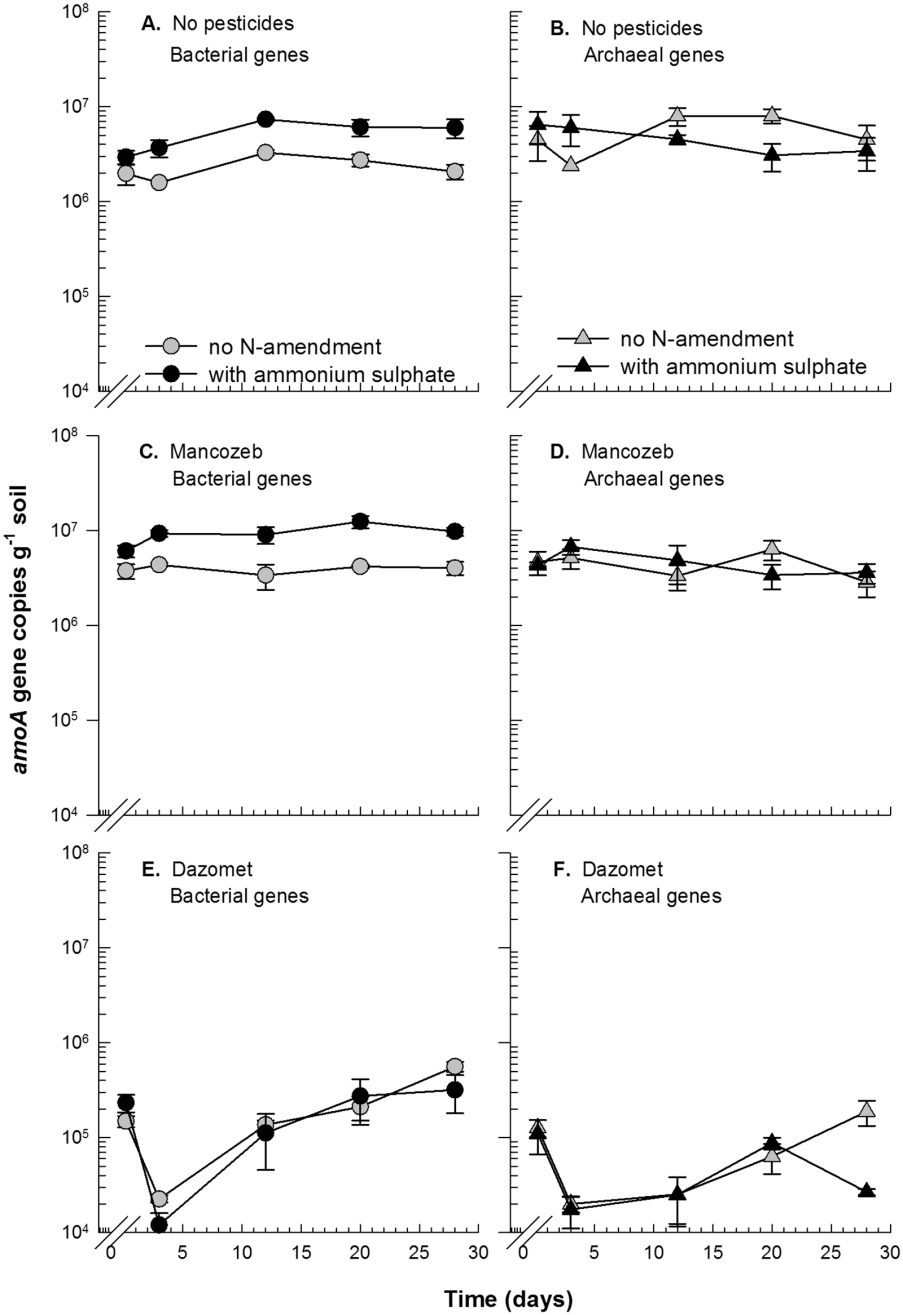
Quantification of *amoA* genes by qPCR. Abundance of *amoA* gene copies for bacteria (A+C+E) and for archaea (B+D+F) in treatments without pesticides (A+B) and in treatments with mancozeb (C+D) and dazomet (E+F). In each plot, the number of genes is shown in treatments without N amendment and with the amendment of ammonium sulfate. The depicted values are the means of triplicate samples, and the error bars indicate standard error. Note that the first data point in each plot indicates measurements one hour after pesticide exposure.

**Fig 3:**
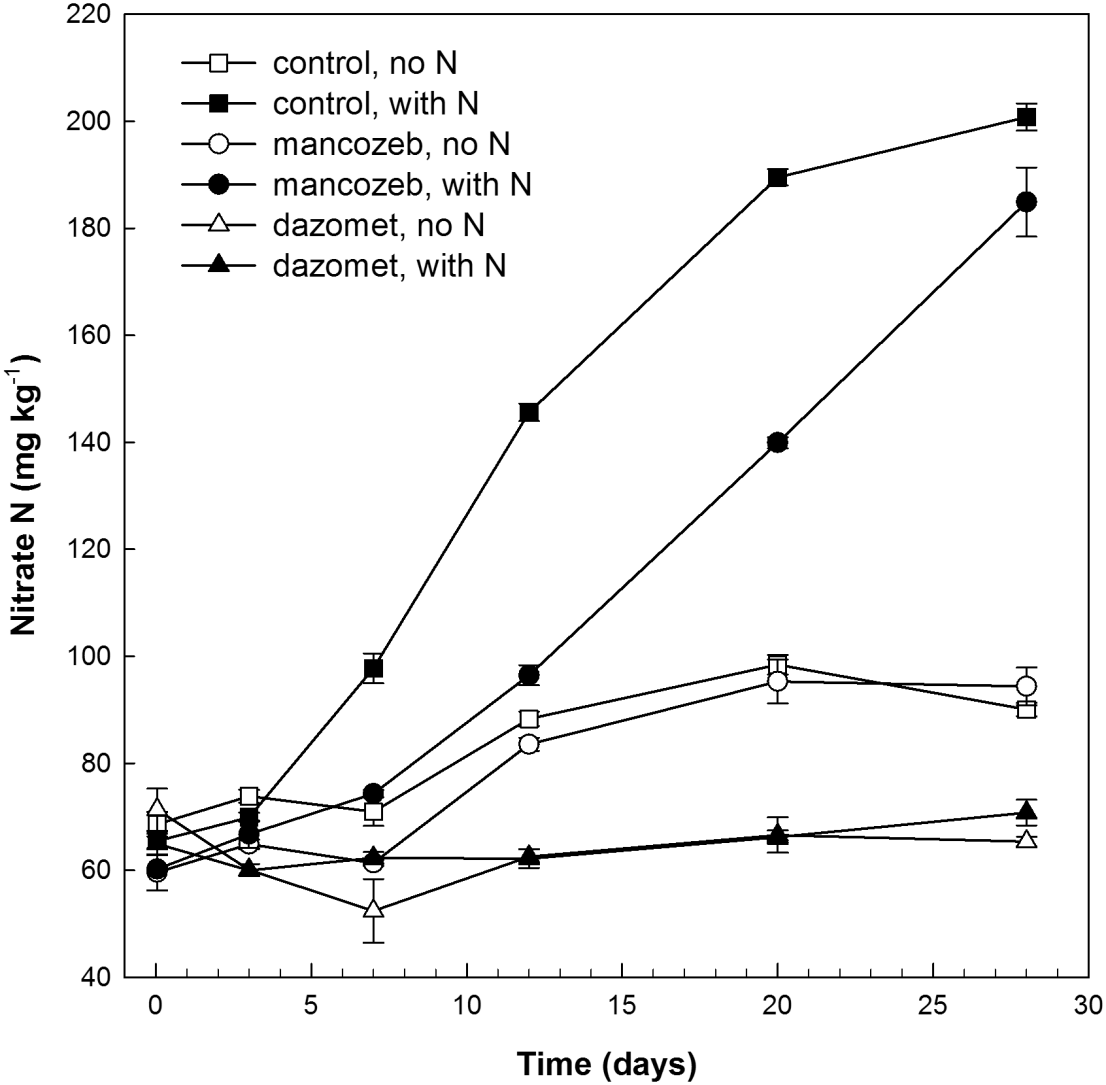
Production of Nitrate. The accumulated production of nitrate is shown in soil without pesticides (squares) and in response to dazomet (triangles) and mancozeb (circles) and in treatments with (filled symbols) and without ammonium sulphate (open symbols).

Treatment with dazomet also reduced the number of *amoA* genes by more than two log units for both the bacteria and archaea (P<0.0001) (Fig 2A, 2B, 2E and 2F). The abundance of *amoA* genes was approximately 2 × 10^6^ copies/g of soil for bacteria and 5 × 10^6^ copies/g of soil for archaea at experimental onset, and these numbers were reduced to approximately 2 × 10^4^ *amoA* gene copies/g of soil three days after treatment. After 28 days, no significant increase in the archaeal *amoA* genes was observed. In contrast, a significant increase in the bacterial *amoA* gene copies showed regrowth of the bacterial ammonia oxidizers in both the N-amended and non-amended soil after dazomet treatment, corroborating the increase in the bacterial *amoA* transcripts. However, the number of *amoA* transcripts and the population size of both bacterial and archaeal ammonia oxidizers were still significantly lower 28 days after treatment compared with the soil without pesticide application. Accordingly, nitrate production was inhibited by dazomet throughout the experiment, and no significant resumption of nitrate production was observed during this 28-day period (Fig 3).

Mancozeb also significantly inhibited nitrate production, particularly in the N-amended soil (P<0.0001) (Fig 3). However, mancozeb did not have a consistent negative impact on the bacterial *amoA* transcripts, which shortly after treatment increased similarly to the control without pesticide application. However, a significant interaction between both time and ammonium sulfate (P<0.001) and mancozeb treatment (P<0.05) indicates that mancozeb conferred inhibition for a limited duration. Three days after treatment, the increase in the bacterial *amoA* transcripts was disrupted, and the maximum abundance of transcripts was not reached until day 21, compared with day twelve in the soil without pesticide (Fig 1A and 1C).

Ammonium sulfate had a positive effect on the bacterial ammonia oxidizers as shown by a significant increase in the bacterial *amoA* gene copies (P<0.001) (Fig 2A and 2C) both in the soil that was treated with mancozeb and in the soil without pesticide application. In contrast, ammonium sulfate had a negative effect on the archaea, as reflected in significant interactions between time and ammonium sulfate (P<0.0001) and mancozeb (P<0.05) on the archaeal *amoA* transcripts (Fig 1B and 1D). The archaeal *amoA* genes, however, were not significantly affected by either mancozeb or ammonium sulfate (Fig 2B and 2D).

### Nitrate production

The nitrate concentration increased rapidly after amendment with ammonium sulfate in the soil without pesticide, and followed a sigmoid trend with an increasing rate, followed by a decreasing rate toward the end of the study (Fig 3). A calculation of the N pools indicates that this development was due to an exhaustion of the added ammonium substrate during the experiment. The accumulated amount of nitrate-N in the N-amended controls (200±4 mg N/kg) approximated the amount of nitrogen that was added to the system as ammonium sulfate plus the nitrate that developed in the non-amended control (190±2 mg N/kg). This suggests that the fraction of produced nitrate-N that was removed by denitrification or biomass incorporation was minor. The nitrate concentration also increased significantly in the non-amended soil during the experiment, albeit approximately seven times less than that in N-amended soil (Fig 3). Because the nitrate-N removal in our systems was minor, we could use our data points to estimate the nitrate production rate as Δnitrate/Δt.

### Correlation between *amoA* abundance and nitrate production

The number of bacterial *amoA* transcripts correlated significantly with the nitrate production rate, albeit with a much higher coefficient of determination in the N-amended (r^2^ = 0.55) than in the non-amended (r^2^ = 0.16) soil (Table 1). The archaeal *amoA* transcripts, however, did not explain the nitrate production rate neither in the N-amended nor in the non-amended soil (r^2^ ≤ 0.13) (Table 1).

**Table 1:** Correlation between nitrate production rates and *amoA* abundance.

*amoA* origin	+(NH_4_)_2_SO4	No N-amendment
Bacterial transcripts	0.55***	0.16*
Archaeal transcripts	0.11^NS^	0.03^NS^
Total transcripts	0.47***	0.11^NS^
Bacterial genes	0.45***	0.18*
Archaeal genes	0.10^NS^	0.13*
Total genes	0.31***	0.16*

Coefficients of determination (r^2^) for the correlation between the nitrate production rates and the number of *amoA* transcripts and genes. The coefficients were determined for the bacterial, archaeal or total summed *amoA* for either N-amended or non-amended treatments. The nitrate production rates were calculated as the net development in nitrate concentration between each sampling day during the experimental period. These rates are plotted against the *amoA* abundance on the last of the respective sampling days. The level of significance is indicated in superscript;

^NS^Non-significant,

*P < 0.05 and

***P < 0.001.

### Quantification of the 16S rRNA and rRNA genes

**Fig 4:**
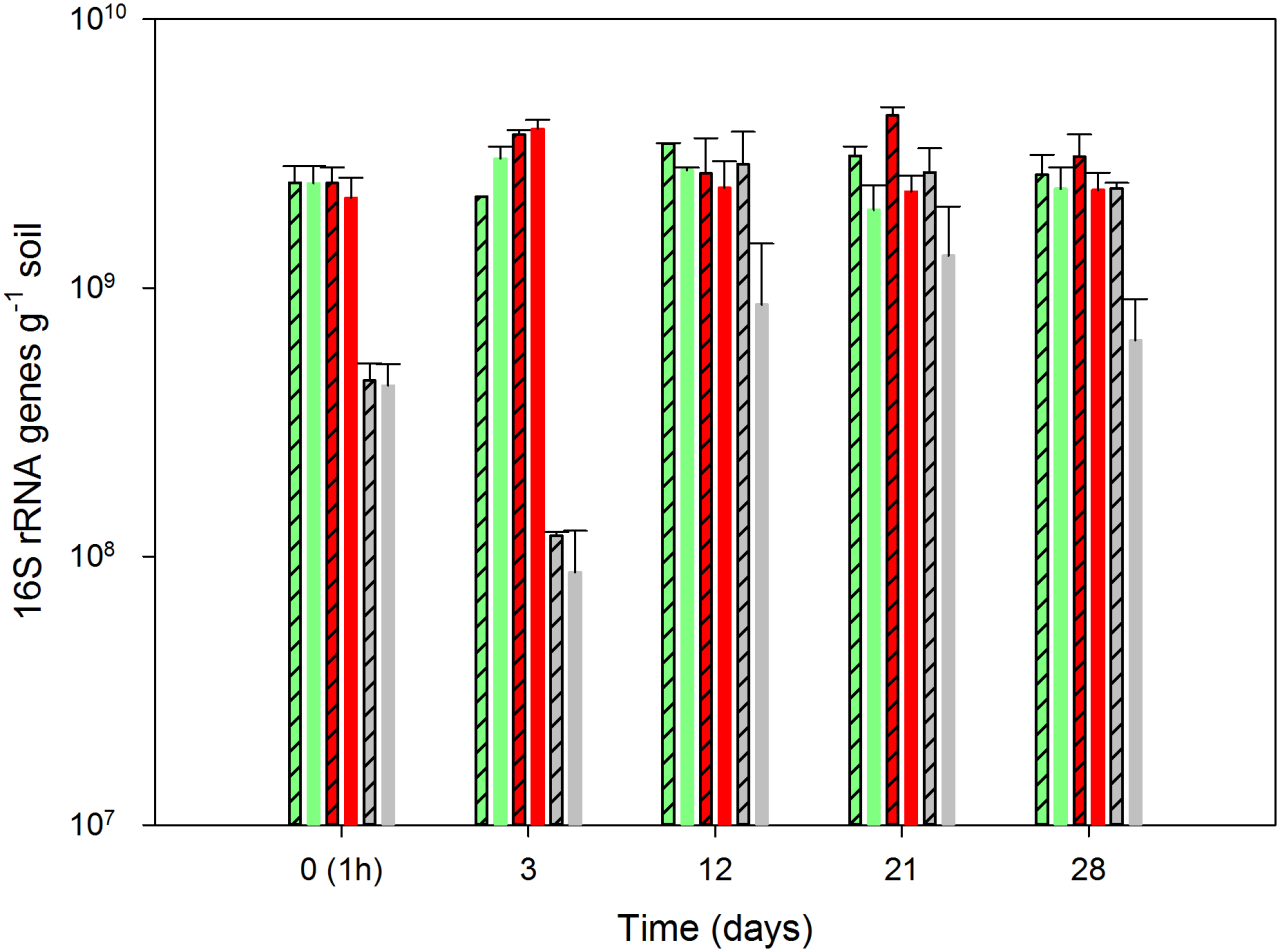
Quantification of total bacteria by qPCR. Abundance of 16S rRNA gene copies is shown in samples from the soil without pesticide (green), with mancozeb (red) and with dazomet (grey). The bars with a crossed pattern represent the non-amended samples, and the filled bars represent the samples that were amended with ammonium sulfate. The depicted values are the means of triplicate samples, and the error bars indicate the standard error.

**Fig 5:**
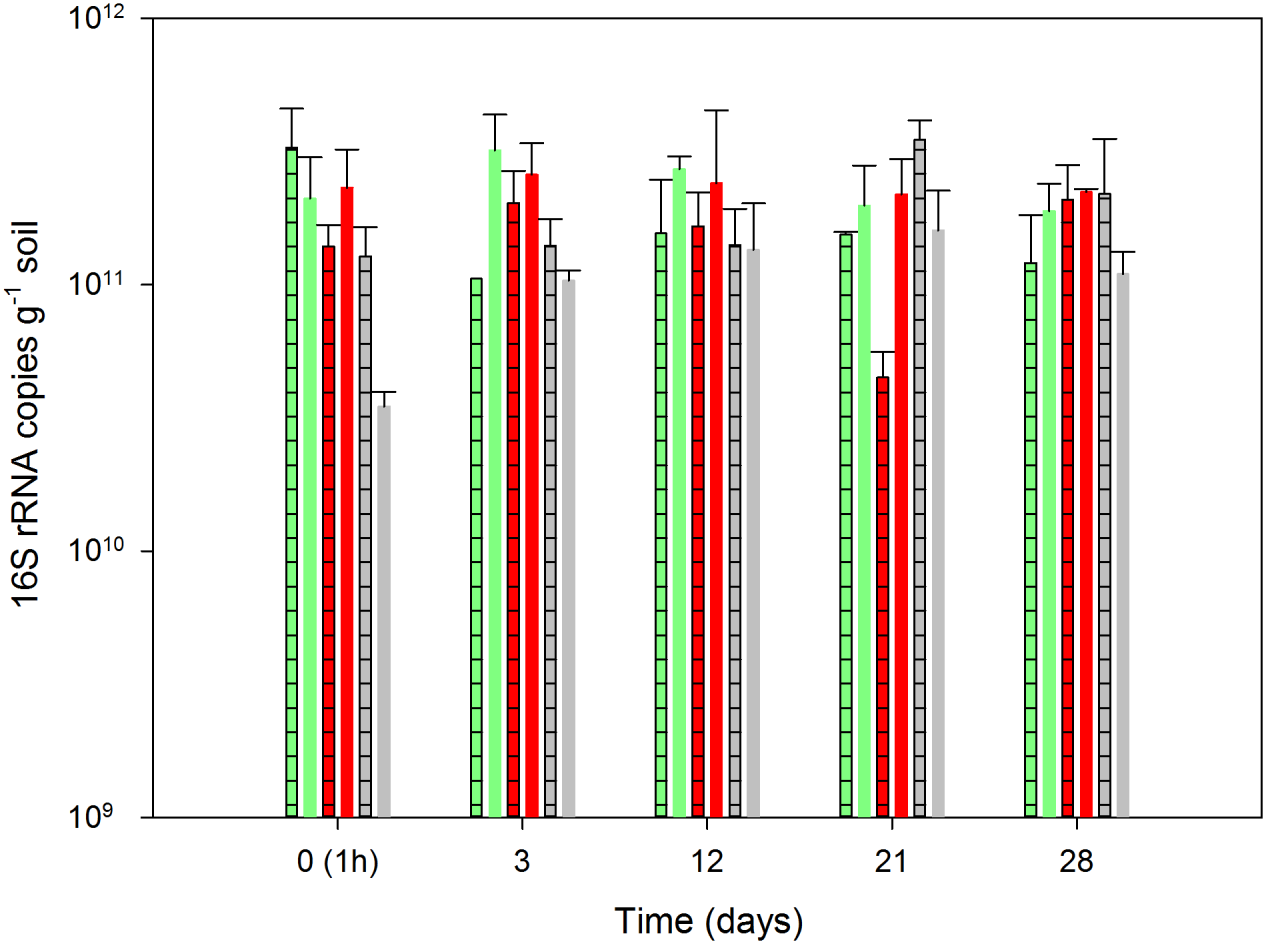
Quantification of total bacteria by qPCR. Abundance of 16S rRNA is shown in samples from the soil without pesticide (green), with mancozeb (red) and with dazomet (grey). The bars with a crossed pattern represent the non-amended samples, and the filled bars represent the samples that were amended with ammonium sulfate. The depicted values are the means of triplicate samples, and the error bars indicate the standard error.

Dazomet significantly reduced the size of the soil bacterial population, and three days after exposure, the number of 16S rRNA genes was reduced by more than one log unit (Fig 4). After twelve days, the 16S rRNA gene number increased again, particularly in the non-amended soil where the population size was fully reestablished. Curiously, however, in the N-amended soil, a significant negative effect of dazomet was still evident after 28 days (Fig 4). The impact of dazomet on the abundance of 16S rRNA, indicating the number of active bacteria in the soil, was short lasting. One hour after the dazomet treatment, the abundance of 16S rRNA decreased in both the N-amended and non-amended soil, but these effects were not significant (Fig 5).

Mancozeb did not have any significant negative effect on the overall number or activity of the bacterial population, except for an unexpected decrease in the 16S rRNA on day 21 (Figs 4 and 5).

### Species richness

**Fig 6:**
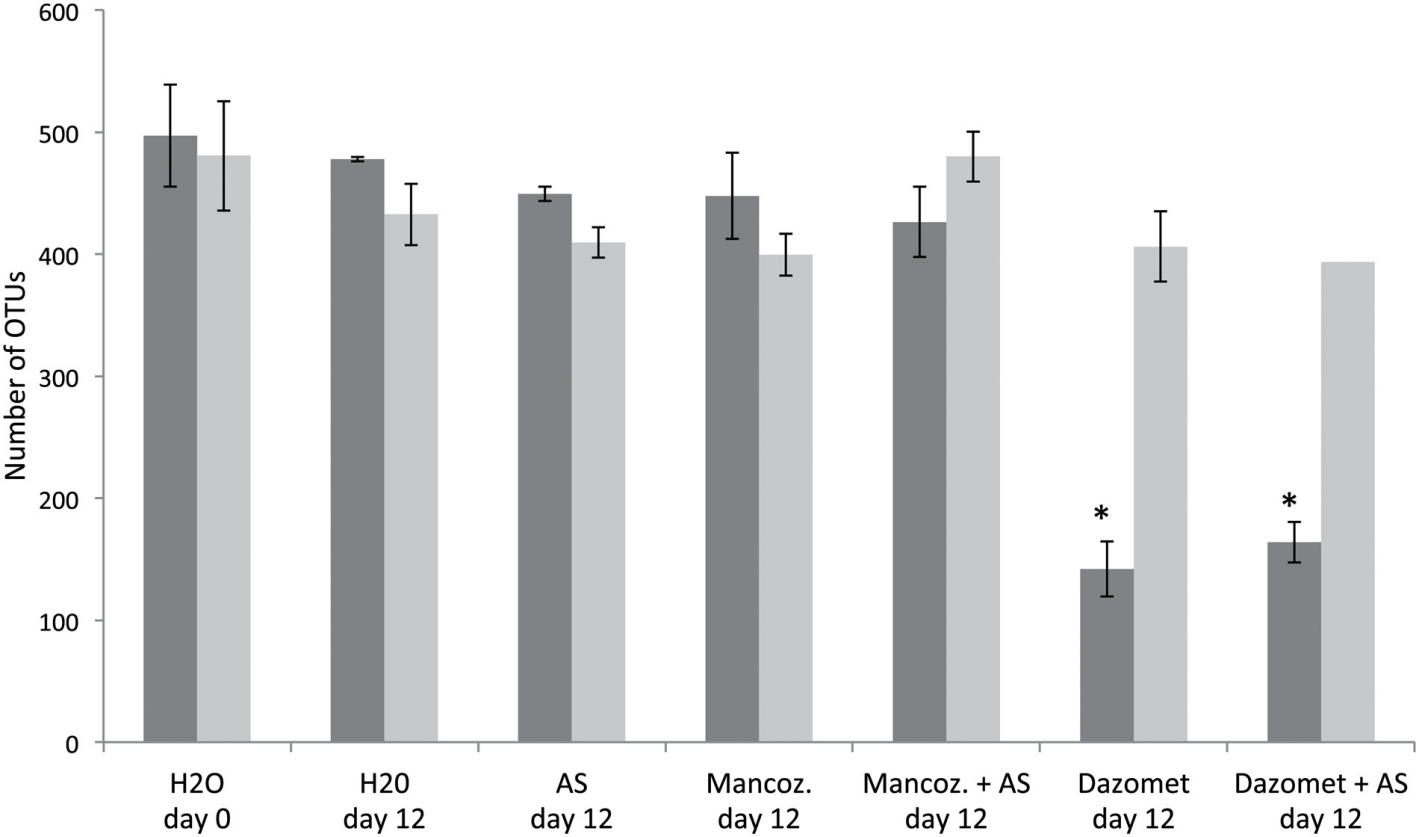
Species richness of total and active bacteria. The figure shows OTUs derived from 454-amplicon sequences of 16S rRNA genes (dark grey bars) and 16S rRNA (light grey bars) representing the control from 1 hour (day 0) and all treatments from day 12. The data presented are the mean and standard deviation of three replicates. Rarefaction analysis was done with 980 randomly selected sequences from each sample. Asterisks represent samples that were statistically different from the non-amended control day 12 (one-way Anova, p < 0.05 followed by Tukey HSD).

**Fig 7:**
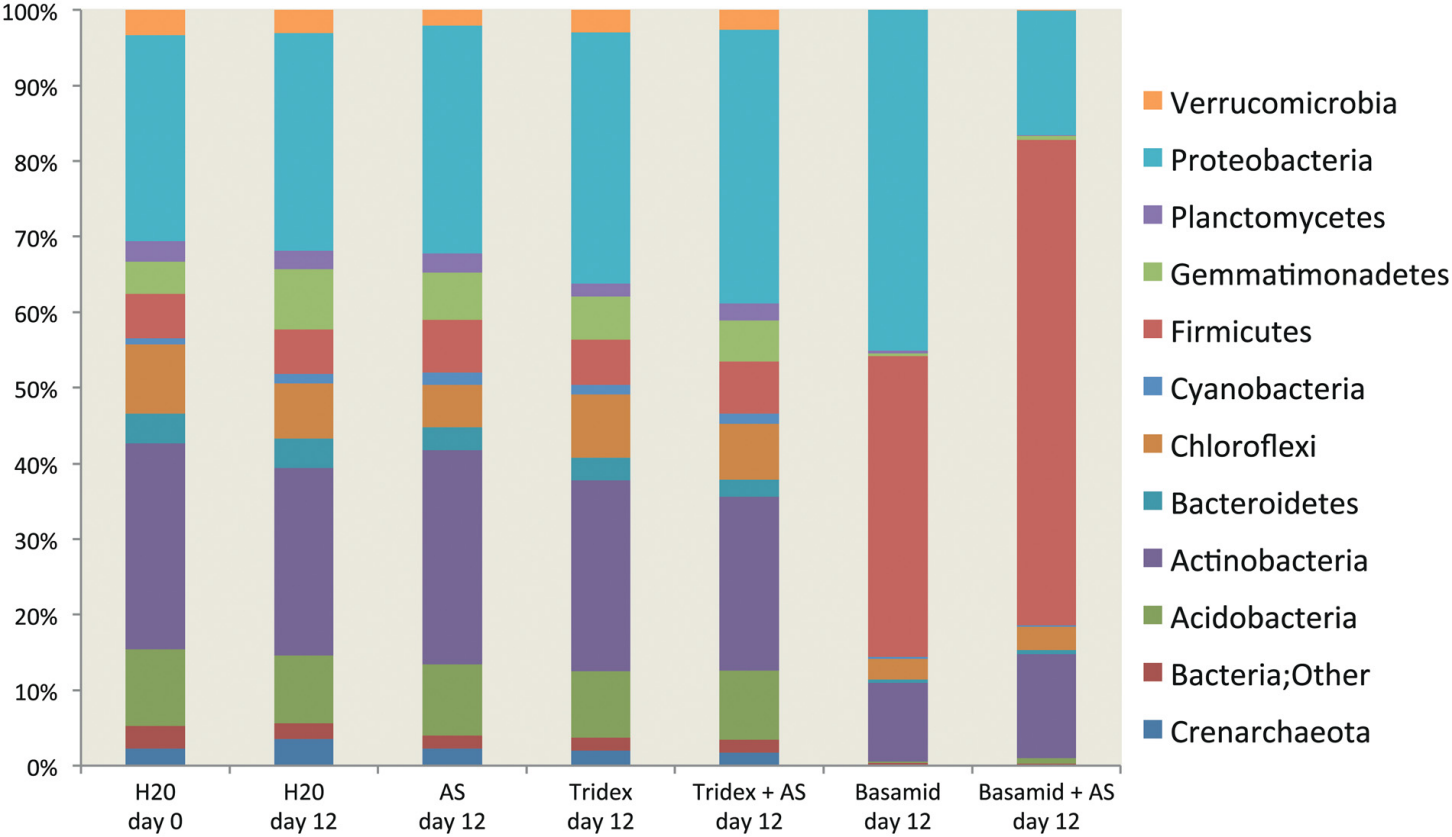
Composition of total bacteria. The bars show relative abundance of the 16S rRNA genes of the twelve most abundant phyla in soil treatments with and without pesticides and with and without ammonium sulfate from day twelve and in the control soil without pesticide and ammonium sulphate from day 0. The bars represent the mean of replicate samples (the individual replicates are shown in S3 Fig).

The identity and number of OTUs were determined for all of the samples from day twelve and for the soil without pesticide application on day 0. The number of OTUs on day twelve was in the range of 400 to 500 for all of the 16S rRNA gene samples, except for those that were treated with dazomet, where OTUs decreased to approximately 150 (Fig 6). In the soil that was treated with mancozeb, the bacterial community composition of the 16S rRNA genes was very similar to that of the soil without pesticide application (Fig 7). Here, Proteobacteria and Actinobacteria were the most abundant and accounted for approximately 25% of the total 16S rRNA gene sequences each, but Firmicutes, Chloroflexi, Acidobacteria and Gemmatimonadetes also occurred in high numbers. Treatment with dazomet resulted in a large increase in the relative abundance, particularly of Firmicutes but also of Proteobacteria, which comprised approximately 85% of the 16S rRNA gene sequences on day twelve. In contrast, the fraction of Sphingomonadales and Rhizobiales decreased upon dazomet treatment. Additionally, the archaeal Crenarchaeota, which are ammonia oxidizers, occurred in low numbers in the control soil but were undetectable in the soil that was exposed to dazomet.

**Fig 8:**
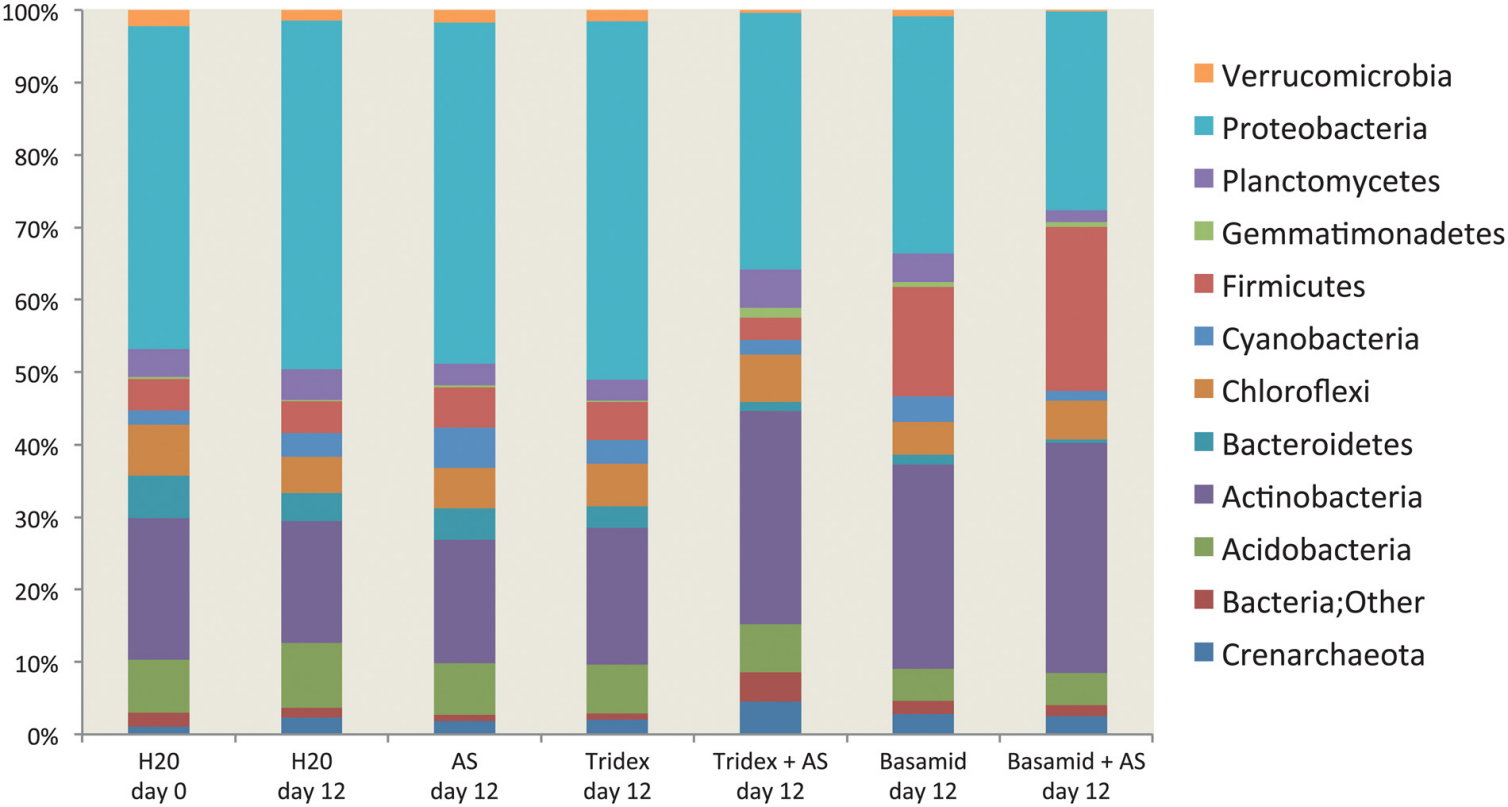
Composition of active bacteria. The bars show relative abundance of the 16S rRNA of the twelve most abundant phyla in soil treatments with and without pesticides and with and without ammonium sulfate from day twelve and in the control soil without pesticide and ammonium sulphate from day 0. The bars represent the mean of triplicate samples.

Although Firmicutes also showed an overrepresentation amongst the active soil bacteria as indicated by the abundance of 16S rRNA sequences (Fig 8), the effect of dazomet treatment on the distribution of the 16S rRNA sequences was lower than the effect on the distribution of the 16S rRNA gene sequences (Fig 7).

### Fate of the pesticides in the soil

The mineralization of mancozeb followed the 1^st^ order of kinetics (R^2^ = 0.99), with the mineralization rate decreasing with time and with the concentration of mancozeb (S1 Fig). The total amount of mancozeb that mineralized after 62 days was 23% without N amendment. Ammonium sulfate significantly (P<0.05) increased the mineralization to 31%.

The sorption of mancozeb in the soil seemed to be determined primarily by the concentration in the aqueous phase. The sorption isotherm could be described by a Freundlich equation with K_F_ values of 1.91 l/kg after two hours of sorption and 2.00 l/kg after 24 hours of sorption (R^2^ = 0.99). The Freundlich constant n that was calculated for the isotherms was 1.04 and 1.01 l/kg for two and 24 hours of sorption, respectively.

The volatilization of the active component of dazomet (MITC) occurred rapidly from the soil under the conditions of water content and temperature corresponding to those that were applied in this experiment. The volatilization measurements showed that approximately 60% of the added MITC evaporated within the first hour and that approximately 95% disappeared from the soil after 48 hours (S2 Fig).

## Discussion

The standard analyses that are used for the hazard assessment of pesticides only provide limited insight into the potentially adverse effects on soil microorganisms. In this study, we focused on two new approaches, including 1) the quantification of *amoA* transcripts as an indicator of the pesticide effects on the activity of ammonia-oxidizing organisms and 2) 454 sequencing as a method to analyze the effect of pesticides on the diversity of soil bacteria.

The impact on the ammonia oxidizers and the bacterial diversity was investigated here in response to the pesticide dazomet, developed to exert a universal pest target effect, and mancozeb, developed to target a more narrow group of pests i.e. fungi. In moist soil, dazomet rapidly transforms into the highly volatile toxicant MITC, which targets biological thiols and amines [39] and is active against bacteria, fungi and larger eukaryotes [40,41]. In this study, dazomet exposure strongly reduced the number of bacterial and archaeal *amoA* transcripts, which was also clearly reflected in the significant inhibition of nitrate production. However, the volatilization experiment with MITC showed that most of this compound evaporated from the soil within three days, and an increase in the *amoA* transcripts occurred. Thus, the reverse-transcription PCR assay targeting *amoA* transcripts was highly sensitive in measuring dazomet inhibition and notably so in measuring the subsequent release of inhibition. Such information on the early recovery was not extractable from the standard nitrification test, most likely due to a lower sensitivity and inability to detect nitrate production from the low number of surviving ammonia oxidizers.

Dazomet exposure also caused a significant decrease in the total number of soil bacteria, but after the evaporation of MITC, the bacterial community resumed growth. The regrowth was primarily due to a confined fraction of resilient and fast-growing bacteria that were largely dominated by members of the order Bacillales and Burkholderiales. *Bacillus* spp. belonging to Bacillales dominates the bacterial community after fumigant exposure, possibly due to the capability to produce stress-resistant endospores [42,43]. A large fraction of bacteria belonging to the Burkholderiales, primarily the family Oxalobacteraceae, are early colonizers with high growth rates [44,45]. Interestingly, we found that the species richness on day twelve after dazomet exposure was higher at the 16S rRNA level than at the gene level (Fig 6). We speculate that this observation is related to a halt in the activity of the dominating early opportunists, which at this stage again became dormant due to the lack of easily accessible carbon sources. Thus, after the growth stagnancy of these few species, the 16S rRNA pool in the soil likely reflects a step toward undisturbed conditions with active bacteria of a broader suite.

Mancozeb inhibits the potential nitrification in arable and grassland soils [46]. In this experiment with mancozeb, we observed a minor inhibition of bacterial *amoA* transcripts in parallel with the inhibition of nitrate production from day three to twelve in the N-amended soil. Curiously, mancozeb did not inhibit bacterial *amoA* transcription immediately after exposure but only after day three. Ethylenethiourea (ETU), which is a degradation product of mancozeb, is a strong inhibitor of nitrification [47]. We hypothesize that the delayed inhibition of *amoA* transcripts may be due to the degradation of mancozeb and the production of the more harmful metabolite ETU in the first few days after exposure [48]. The mineralization and sorption experiments for mancozeb indicate that the majority of mancozeb had either been fully degraded through ETU and EU to carbon dioxide or sorbed into the soil after approximately two weeks. We propose that the bioavailability of ETU in particular was negatively correlated with bacterial *amoA* transcription and nitrate production, resulting in a shorter inhibition period of the pesticide.

Ammonia-oxidizing bacteria, due to high sensitivity, have often been used as indicator organisms to monitor soil perturbations. The quantification by qPCR or fingerprinting by DGGE or T-RFLP of *amoA* genes or specific 16S rRNA genes of ammonia oxidizers have been applied to assess specific changes in the population [49–52]. However, many of these studies are indiscriminately based on DNA, which may largely derive from functionally inactive bacteria. Instead, focus on the active portion of the population is likely to result in a stronger response, e.g., Nyberg and co-workers found that 4-ethylphenol affected the community composition of actively replicating ammonia oxidizing bacteria but not that of the inactive population [53].

In this study, the development in bacterial *amoA* transcript number correlated with nitrate production and with the *amoA* gene numbers when ample substrate was available. Therefore, under these conditions, the conversion of ammonia by ammonia monooxygenase is tightly connected to growth, thereby linking *amoA* transcripts, nitrate production and *amoA* genes. In this N-amended system, both bacterial *amoA* transcripts and *amoA* genes could be used as indicators of pesticide inhibition, although transcript numbers responded more promptly and showed a slightly better correlation with nitrate production compared with the *amoA* genes.

In the non-amended soil, the correlation between the bacterial *amoA* transcripts and nitrate production was very low compared with the N-amended soil. We speculate that substrate depletion in the non-amended soil limited the bacterial ammonia oxidizers and allowed archaea to take over a larger part of nitrification. In this system, pesticide application also inhibited bacterial *amoA* transcripts, but concomitant substrate limitation seemed to blur the release of inhibition, thereby making it more difficult to assess the impact of the pesticides.

Several studies have stated that bacteria rather than archaea are the major ammonia oxidizers in N-rich soil ecosystems. Although this statement has been accepted as a general phenomenon, the reason for this niche differentiation between communities is still greatly debated [14,54,55]. Jia and Conrad demonstrated that nitrification parallels the abundance of bacterial but not archaeal *amoA* gene copy numbers in soil microcosms that are amended with ammonium sulfate [56]. Likewise, nitrate concentration correlated (R^2^ = 0.56) with bacterial but not archaeal *amoA* gene copies in six grassland soils that were amended with urine-N substrate [57]. In fact, the abundance of archaeal *amoA* genes was negatively related to N-fertilization in both of these studies. We similarly observed that N amendment resulted in a 1-log decrease in archaeal *amoA* transcripts and a tendency to decrease in the archaeal *amoA* gene copies. In contrast, the archaeal *amoA* dominated in the non-amended soil. Despite the high abundance, the number of archaeal *amoA* transcripts or genes did not show any correlation with nitrate production in this study. The lacking correlation of nitrate production with archaeal *amoA* in general and the weak correlation with bacterial *amoA* genes under low substrate availability could be partly explained by mixotrophic growth [56,58]. Furthermore, a change in the conversion factor between the transcription of *amoA* and the activity of the ammonia monooxygenase may occur in response to altered substrate availability. Such a switch may be present in the bacterial ammonia oxidizers *Nitrosospira briensis* and *Nitrosomonas europaea*, for which both a constitutive and a higher substrate-induced level of ammonia oxidation have been reported [59,60]. A deeper knowledge of this regulation is required to understand ammonia oxidation under N-limiting conditions.

We believe that molecular techniques targeting particularly the active soil microbial community can be applied to broaden the current hazard assessment of pesticide side effects. In this study, we demonstrated that the sequencing of the 16S rRNA and the quantification of the *amoA* transcripts provide valuable input to assess the impact on the soil microbial community. Similar to other process-based methods, this molecular approach is more sensitive when care is taken to avoid the inhibition of microbial activity by other stress factors, such as substrate limitation. We recommend bacterial *amoA* transcripts as good bioindicators of pesticide inhibition that, when substrate is provided, can also be used to measure ammonia oxidation.

## Supporting Information

S1 Fig Mineralisation of mancozeb. Accumulated mineralisation curves for mancozeb at 20°C in soil depicted as the percentage of added pesticide. Mineralisation of mancozeb without N-amendment is shown as filled squares and mancozeb with ammonium sulphate as open squares. Error bars show standard error of the means.
(TIF)

S2 Fig Dazomet evaporation from soil. Volatilization of the active component of dazomet (MITC) from soil at 20°C and 60% WHC.
(TIF)

S3 Fig Total bacterial composition. Relative abundance of the twelve most abundant phyla for individual replicates A-C of the control samples without pesticide or ammonium sulphate from day 0 and for all treatments at day 12. The dazomet treated samples only exist in duplicate because of problems with the nucleotide quality.
(EPS)
